# Reversible cerebral vasoconstriction syndrome: strategies to early diagnosis and the role of transcranial color-coded doppler ultrasonography (TCCD)

**DOI:** 10.1007/s10072-023-06755-3

**Published:** 2023-04-04

**Authors:** Nicola Merli, Marina Padroni, Cristiano Azzini, Andrea Bernardoni, Carla Marcialis, Valeria Tugnoli, Vincenzo Inchingolo, Maura Pugliatti

**Affiliations:** 1 Department of Neuroscience and Rehabilitation, University of Ferrara, Ferrara, Italy; 2 Department of Neuroscience and Rehabilitation, S. Anna University Hospital, Ferrara, Italy; 3 Department of Radiology, S. Anna University Hospital, Ferrara, Italy; 4 Interdepartmental Research Center for Multiple Sclerosis and Other Inflammatory and Degenerative Disorders of the Nervous System, University of Ferrara, Ferrara, Italy; 5 Neurology Unit, Fondazione IRCCS Casa Sollievo Della Sofferenza, San Giovanni Rotondo, Italy

**Keywords:** Reversible cerebral vasoconstriction syndrome, Transcranial color-coded Doppler, Thunderclap headache, Cerebrovascular diseases

## Abstract

**Background:**

Reversible cerebral vasoconstriction syndrome (RCVS) is a cerebrovascular transitory condition characterized by severe headache, possible concomitant acute neurological symptoms, evidence of diffuse multifocal segmental constriction of cerebral arteries, and usually spontaneously resolving within 3 months. Putative causes and/or precipitating factors are vasoactive drugs—e.g., antidepressants, α-sympathomimetics, triptans—post-partum, and immunosuppressants.

**Case presentation:**

We report the case of a middle-aged woman referred to the emergency room (ER) with a 7-day long intense headache and vomit. Cerebral non-contrast computed tomography (CT) was negative for acute ischemic lesions or intracranial bleedings. She was again referred to ER 7 days later with additional fluctuating episodes of weakness in left arm and both lower limbs. A new brain CT was negative. Due to worsening headache, a transcranial color-coded Doppler (TCCD) was performed, which showed diffuse multifocal blood flow acceleration in all principal intracranial vessels, and particularly on the right hemisphere. These findings were subsequently confirmed at MR angiogram and digital subtraction angiography.

**Conclusion:**

TCCD imaging is a non-invasive and relatively inexpensive tool which provides real-time information on cerebrovascular function, blood flow velocities, and hemodynamic changes. TCCD may be a powerful tool in the early detection of acute infrequent cerebrovascular conditions, as well as in monitoring their course and the therapeutic response.

A 54-year-old Caucasian woman was referred to the emergency room (ER) on October 21, 2021, with intense headache and vomiting dating 7 days back. Her clinical history included major depressive disorder treated with venlafaxine, fibromyalgia, and hypothyroidism.

Brain computed tomography (CT) was negative for acute ischemic lesions or intracranial bleedings. Neurological examination excluded meningeal irritation or focal deficits, and she was discharged from ER with headache ascribed to fibromyalgia.

She was referred to ER again 7 days later with persistent intense headache and fluctuating episodes of weakness in left arm and lower limbs. Clinical neurological examination revealed moderate left hemiparesis. A new brain CT scan was normal. The patient was admitted to the Neurology ward in observation. In the suspect of transient ischemic attacks, acetylsalicylic acid was started and a prompt improvement of motor deficits followed.

On day 3, she complained a remarkable re-exacerbation of headache and malaise, not elicited by any specific trigger event or effort. Furthermore, she reported having experienced thunderclap headache (TCH) during physical exertion 2 weeks before first ER admission. Headache irradiated from occipital to apex region with constrictive and throbbing features, worsened by intense Valsalva manoeuvre, and fluctuated over the next days.

A transcranial color-coded Doppler (TCCD) performed on the same day showed diffuse bilateral multifocal blood flow acceleration in all principal intracranial vessels, especially in the right hemisphere, with focal *aliasing* at color-Doppler signal and low flow turbulences (Fig. 1). Lindegaard index was greater than 7 on the right and 4.5 on the left side, indicating vasospasm. Interestingly, the greater involvement of right ACM M1 and M2 segments at TCCD was consistent with clinical (left hemiparesis) and brain magnetic resonance imaging (MRI) (subarachnoid haemorrhage (SSH) at right cortical frontal convexity).

**Fig. 1:**
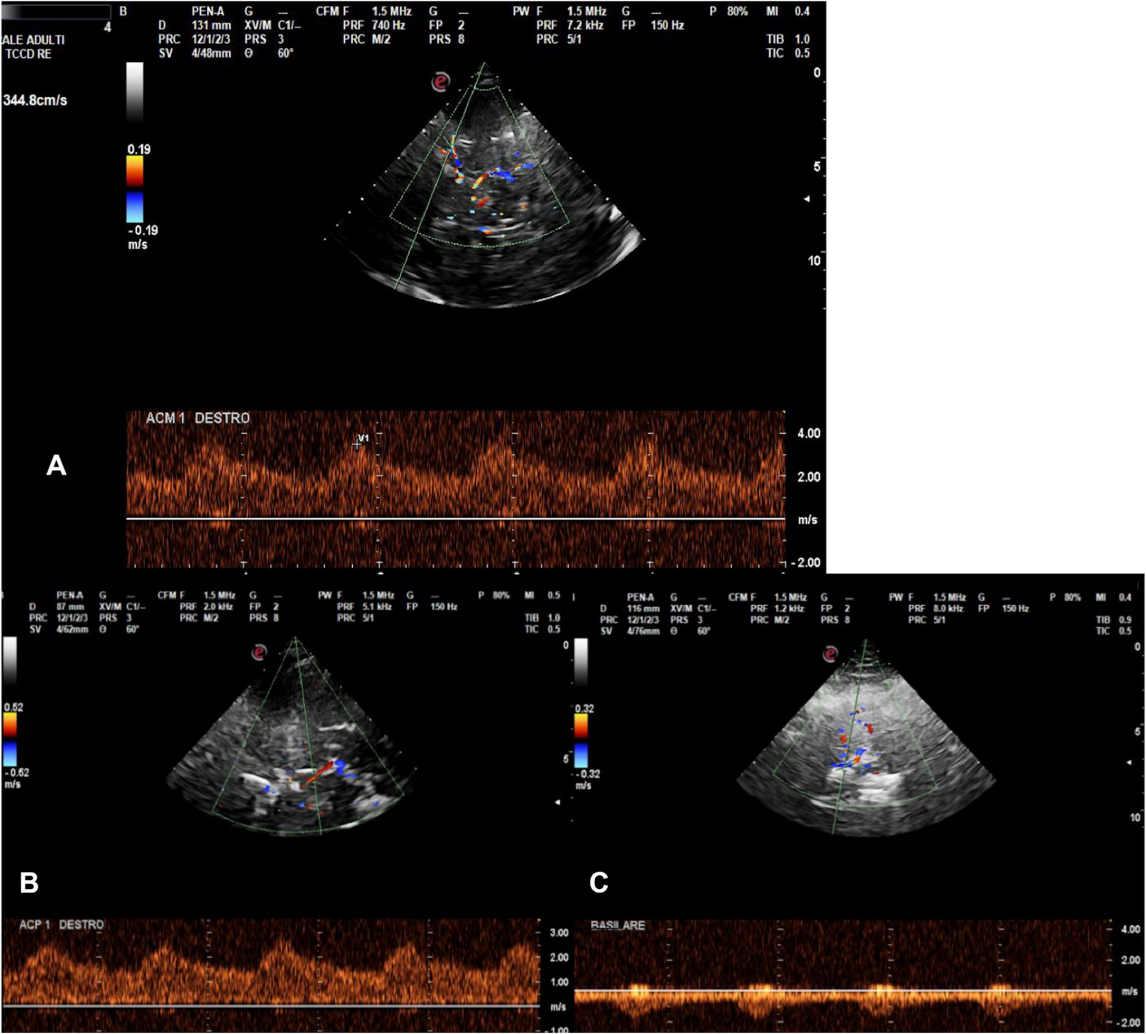
Transcranial color-coded Doppler (TCCD) images showing focal acceleration, diffuse aliasing, and flow turbulence in the principal intracranial vessels. **A** Right distal middle cerebral artery (M1) with peak systolic velocity (PSV) of ≈ 380 cm/s, **B** right posterior cerebral artery (P1) with PSV ≈ 300 cm/s, and **C** basilar artery with PSV ≈ 180 cm/s and flow turbulence

Laboratory investigations for autoimmune disease were all normal. Subsequently performed brain MRI and MR angiogram (MRA) documented diffuse and multifocal narrowing and irregularities of intracranial vessels (Fig. 2). A thin cortical convexity SAH and two restricted-diffusion areas indicating small recent ischemic lesions were also detected with MRI.

**Fig. 2:**
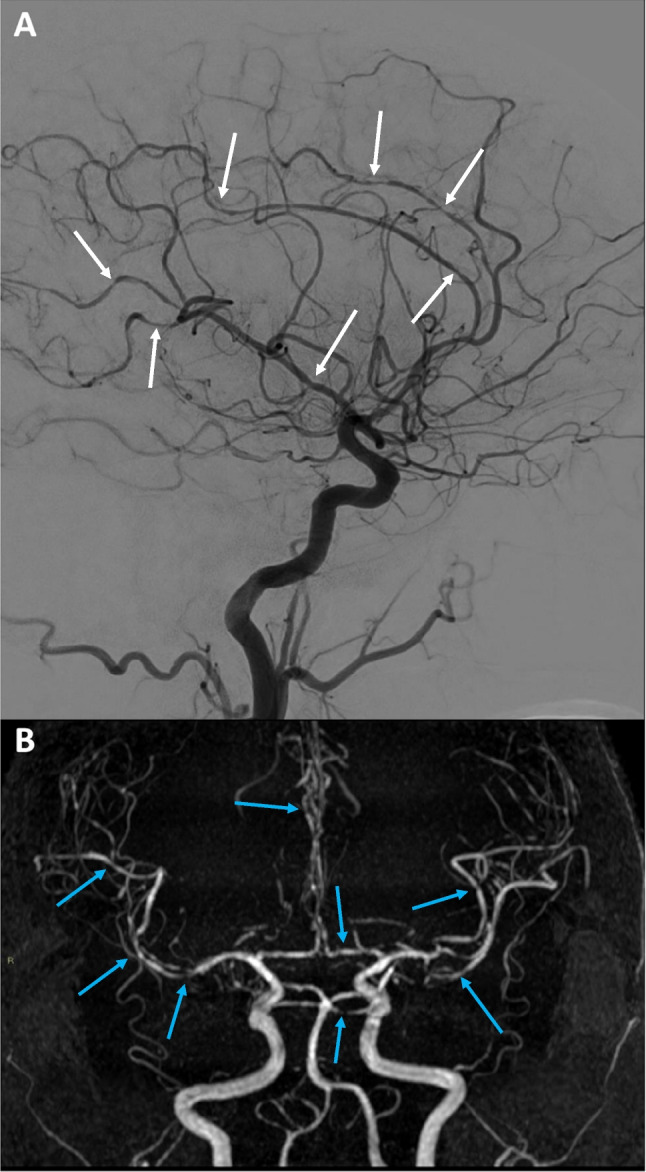
Cerebral angiography (**A**) and magnetic resonance angiography (MRA) (**B**) depicting remarkable smooth multifocal widespread narrowing and irregularities in the vessels of both anterior and posterior intracranial circulation

Finally, digital subtraction angiography (DSA) was compatible with multifocal vasoconstriction of middle, anterior, pericallosal, and posterior cerebral arteries of both sides (Fig. 2), while vascular malformations or saccular aneurysms were ruled out. Based on clinical history and instrumental findings, a diagnosis of reversible cerebral vasoconstriction syndrome (RCVS) was made.

The patient was efficaciously treated with nimodipine 60 mg every 4 h, showing a gradual decrease in intracranial blood flow velocities at TCCD already during the first week of treatment as well as remission of neurological symptoms. Neurological assessment after 3 months from hospital discharge confirmed significant decrease of velocitometric parameters and vasospasm resolution, supported by disappearance of fluctuating motor deficits and complete clinical recovery.

## Discussion

RCVS is a rare, likely underdiagnosed, neurologic disorder, which currently gains interest in relation to an unceasing growth of diagnostic acquisitions and technology of cerebrovascular imaging. RCVS pathophysiology remains unclear. Sympathetic overactivity and dysregulation of vascular tone in association with specific triggering conditions, and blood–brain-barrier breakdown with the presence of endothelial dysfunction have been hypothesized to generate vasoconstriction [1–3]. A broad spectrum of exogenous factors has been related to RCVS. The pathogenetic role of sympathetic overactivity is supported by association with the use of adrenergic and vasoactive substances (e.g., cocaine, ecstasy, amphetamines, marijuana). Medications, such as antidepressants (selective serotonin re-uptake inhibitors or SSRI, noradrenaline, and serotonin re-uptake inhibitors or NSRI), triptans, and nasal decongestants with sympathomimetic properties may also trigger RCVS. Other such mechanisms include physical exercise (sport), conditions implying Valsalva manoeuvre (e.g., defecation, coughing, sneezing), sexual activity or emotional situations, late pregnancy, early puerperium, pre-eclampsia and eclampsia, pheochromocytoma, hypercalcemia, and catecholamine-secreting tumors [4].

RCVS is associated with a wide variety of clinical and radiological manifestations. Neuroimaging reveals multifocal and segmental cerebral vasoconstriction in more than two branches of the circle of Willis [5]. RCVS usually exhibits a monophasic course and sudden presentation with pathognomonic TCH. Headache is typically bilateral with occipital onset; it can re-exacerbate spontaneously and in association with transient or persistent focal deficits. RCVS neurological manifestations may also include hemiparesis or hemiplegia, aphasia, hemianopia, ataxia, photophobia or phonophobia, nausea and emesis, and seizures [6, 7].

Neuroradiological imaging is reported as the gold standard for detecting abnormalities of cerebral vessels caliber. However, because of the dynamic process underlying RCVS, brain MRI may not be sensitive enough to detect dynamic abnormalities at an early disease stage, but also early signs of convexity SAH, superficial border-zone infarcts, PRES, and lobar hemorrhages, usually detectable over a few days or weeks [6, 8].

Angiographic imaging shows relatively smooth, proximal, bilateral, and rather symmetric multisegmental vessel narrowing, involving both the posterior and anterior circulation with the typical *sausage-on-a-string* aspect. Of note is the widespread dynamic nature of vasospasm, exhibiting vasoconstriction resolution for some vessels and activation in others in the ensuing days [8, 9].

Diagnostic criteria of RCVS also include normal cerebrospinal fluid (CSF) analysis, absence of aneurysmal subarachnoid hemorrhage, and substantial regression of vasoconstriction within 3 months [1, 4, 6].

TCCD is a non-invasive ultrasonographic tool easily providing relevant information on intracranial hemodynamics. In RCVS, TCCD allows for detecting vasospasm even at an early stage and for monitoring the RCVS dynamic nature. Specifically in our case who featured a misleading presentation of RCVS, TCCD performed during the early phase (i.e., headache) was diriment to detect early signs of multifocal vasospasm and its propagation to large vessels, even prior to MRI. TCCD proved an efficient and effective non-invasive tool to detect RCVS at an early stage, preciously contributing to decisional support in differential diagnosis as well as in the monitoring of vasospasm changes and pharmacological response.

### RCVS: take home points

- Peculiar presentation with sudden thunderclap headache, most often in women aged 20–50 years
- Typically associated triggers are physical exertion, Valsalva maneuver, vasoactive drugs or medications, complicated pregnancy, and puerperium
- MRI can be normal at an early stage and show SAH, watershed infarcts, or PRES after a few days from onset.
- MRA and DSA studies show *sausage-on-a-string* morphology (smooth tapered narrowing and dilatation)
- No evidence of aneurysmal SAH
- Neurosonology (TCCD): uni- or bilateral multifocal blood flow acceleration in more than one vessel, diffuse low frequency turbulence at Doppler spectrum, multifocal areas of aliasing at color-Doppler; Lindegaard Index > 3 confirming vasospasm

## References

[1] Ducros A, Wolff V (2016). The typical thunderclap headache of reversible cerebral vasoconstriction syndrome and its various triggers. Headache.

[2] Cho S, Ling YH, Lee MJ, Chen SP, Fuh JL, Lirng JF, Cha J, Wang YF, Wang SJ, Chung CS (2020). Temporal profile of blood-brain barrier breakdown in reversible cerebral vasoconstriction syndrome. Stroke..

[3] Lee MJ, Cha J, Choi HA, Woo SY, Kim S, Wang SJ, Chung CS (2017). Blood–brain barrier breakdown in reversible cerebral vasoconstriction syndrome: implications for pathophysiology and diagnosis. Ann Neurol.

[4] Calabrese LH, Dodick DW, Schwedt TJ, Singhal AB (2007). Narrative review: reversible cerebral vasoconstriction syndromes. Ann Intern Med.

[5] Cappelen-Smith C, Calic Z, Cordato D (2017). Reversible cerebral vasoconstriction syndrome: recognition and treatment. Curr Treat Options Neurol.

[6] Ducros A (2012). Reversible cerebral vasoconstriction syndrome. Lancet Neurol.

[7] Caria F, Zedde M, Gamba M, Bersano A, Rasura M, Adami A, Piantadosi C, Quartuccio L, Azzini C, Melis M, Luisa Delodovici M, Dallocchio C, Gandolfo C, Cerrato P, Motto C (2019). The clinical spectrum of reversible cerebral vasoconstriction syndrome: the Italian Project on Stroke at Young Age (IPSYS). Cephalalgia.

[8] Singhal AB, Topcuoglu MA, Fok JW, Kursun O, Nogueira RG, Frosch MP, Caviness VS (2016). Reversible cerebral vasoconstriction syndromes and primary angiitis of the central nervous system: clinical, imaging, and angiographic comparison. Ann Neurol.

[9] De Boysson H, Parienti JJ, Mawet J, Arquizan C, Boulouis G, Burcin C, Naggara O, Zuber M, Touzé E, Aouba A, Bousser MG, Pagnoux C, Ducros A (2018). Primary angiitis of the CNS and reversible cerebral vasoconstriction syndrome A comparative study. Neurology.

